# Supplementary material for: Complexome profiling of the Chlamydomonas psb28 mutant reveals TEF5 as an early PSII assembly factor
Source: Plant Cell. 2025 Mar 17;37(6):koaf055. doi: 10.1093/plcell/koaf055 (PMC12164590; doi:10.1093/plcell/koaf055)
Supplement: koaf055_Supplementary_Data [file koaf055_supplementary_data.zip › psb28_interactive.zip!/plots/intensity/qualityPlots/peptides/psaJ_Cre-1.g2716958.t1.p1.html]

### Cre-1.g2716958.t1.p1

Trivial name: psaJ  
SeqCoverage [median]: 24.40  
Mass: 4.75 kDa  
  
 

| Sequence | MissedCleavage | Mass [Da] | wtIntensity | muIntensity | Score |
| --- | --- | --- | --- | --- | --- |
| YFPDPLVFSF | 0 | 1230.60 | 175171.12 | 52782.97 | 156.40 |
|  |  |  | 175171.1 | 52783.0 |  |
